# Effect of cataract surgery on the progression of age-related macular degeneration

**DOI:** 10.1097/MD.0000000000031566

**Published:** 2022-11-04

**Authors:** Zhaoyan Chen, Ya Zeng, Fangyuan Tian

**Affiliations:** a Department of Pharmacy, West China Hospital, Sichuan University, Sichuan, PR China.

**Keywords:** age-related macular degeneration, cataract, meta-analysis, surgery, systematic review

## Abstract

Cataracts and age-related macular degeneration (AMD) are common causes of decreased vision and blindness in individuals over age 50. Although surgery is the most effective treatment for cataracts, it may accelerate the progression of AMD, so this study further evaluated the influence of cataract surgery for AMD through a systematic review and meta-analysis.

**Methods:**

The Cochrane Systematic Evaluation method was adopted, and computer searches were conducted for the China Knowledge Network, Wanfang, Vipul, SinoMed, PubMed, SpringerLink, Clinicalkey, Medline, Cochrane Library, Web of Science, OVID, and Embase databases of cohort studies on the impact of cataract surgery on AMD, with search timeframes up to May 2022. Meta-analysis was performed using Stata/12.0.

**Results:**

A total of 8 cohort studies were included in the study. The results showed that the relative risk (RR) of AMD progression after cataract surgery was not significantly different, RR 1.194 [95% credibility interval (CI) 0.897‐1.591]; the risk remained increased more than 5 years after surgery, RR 1.372 (95% CI 1.062‐1.772).

**Conclusion:**

There is still a significant positive correlation between cataract surgery and increase the risk of worsening of AMD progression, and faster progression of early-to-late AMD found in cataract surgery with longer follow-up of patients.

## 1. Introduction

Cataracts and age-related macular degeneration (AMD) are common causes of decreased vision and blindness in individuals over age 50.^[1]^ By 2020, the number of people with AMD globally is expected to be approximately 200 million, increasing to nearly 300 million by 2040.^[2]^ For the past few decades, vision has been an important criterion for cataract surgery, and cataracts can improve vision by removing the opaque lens.^[3]^ Surgery is the most effective operation in cataract patients to improve visual function, but some professor’s suspect it increase the risk of worsening of underlying AMD and vision.^[3,4]^ This potential problem has not been resolved for a long time. Prospective or retrospective studies^[5,6]^ have reported a higher frequency of AMD progression in surgical eyes than in nonoperated eyes. However, some studies^[7–11]^ have also shown that there is no significant correlation between cataract surgery and AMD. A cross-sectional^[12]^ study also explored this topic, but it is still inconclusive. The truth is that cataracts and AMD often coexist, and there may be adverse effects between AMD and cataract surgery. However, delaying cataract surgery can also negatively impact a patient’s vision. Therefore, this study further evaluated the influence of cataract surgery for AMD through a systematic review and meta-analysis.

## 2. Materials and Methods

### 2.1. Search strategy

A meta-analysis was performed according to the PRISMA guidelines.^[13]^ We independently searched PubMed, SpringerLink, Clinicalkey, Medline, the Cochrane library, Web of Science, OVID, Embase, and SinoMed from their earliest dates up to May 2022. The final search string was “macular degeneration,” “wet macular degeneration,” “choroidal neovascularization,” “geographic atrophy,” “age-related macular degeneration” and “randomized controlled trial.”

### 2.2. Inclusion criteria

Cohort study comparing visual acuity in AMD patients with or without cataract surgery.

### 2.3. Research objects

Age-related cataract patients who underwent cataract emulsification surgery or suffered from both cataracts and AMD were included. Those who had cataract history may affect postoperative visual acuity and were therefore excluded.

### 2.4. Outcome measures

To describe whether cataract surgery increases the risk of worsening of underlying AMD and vision (relative risk (RR)).

### 2.5. Data extraction

For each study, 2 reviewers independently evaluated the retrieved literature against the inclusion and exclusion criteria. In case of disagreement, adjudicate by consultation or with the aid of a 3rd commentator. To ensure the consistency of the data collection of each study, we conform to the conditions of the study of the following information into a structured Excel data table: first author, year of publication, study design, number of control and case groups, location, patients’ age, AMD classification, duration of follow-up, RR, odds ratio (OR) and hazard ratio (HR) with corresponding 95% CI [credibility interval (CI)]. The one that adjusts the most variables was used when there were multiple estimates.

### 2.6. Quality evaluation and statistical analysis

The risk of bias of nonrandomized studies was assessed using the Newcastle-Ottawa scale (NOS).^[14]^ In consideration of the low prevalence of AMD, the distinction between RR/OR/HR can generally be ignored.^[15]^ We used Stata 12.0 to derive pooled effect estimates such as risk ratios (RR) using a fixed-effects model or a random-effects model.^[16]^ Heterogeneity test: The *χ*^2^ test was used to analyze the statistical heterogeneity between studies with an α level of 0.1, and the magnitude of heterogeneity was quantitatively estimated according to *I*^2^. If there was statistical heterogeneity between studies (*P* > .1 and *I*^2^ < 50%), a fixed effect model was used; if *P* < .1 and *I*^2^ > 50%, but when it was clinically judged that the research indicators of each group were consistent and needed to be merged, a random effect model was selected to merge the effect values.^[15]^ We use sensitivity analyses to assess the impact of individual studies on the outcome, funnel plots quantified by Egger and Begg tests to observe potential publication bias, and region, duration of follow-up, and AMD classification to performed subgroup analyses. *P* < .05 was considered statistically significant.

## 3. Results

### 3.1. Literature search

**Figure 1.**
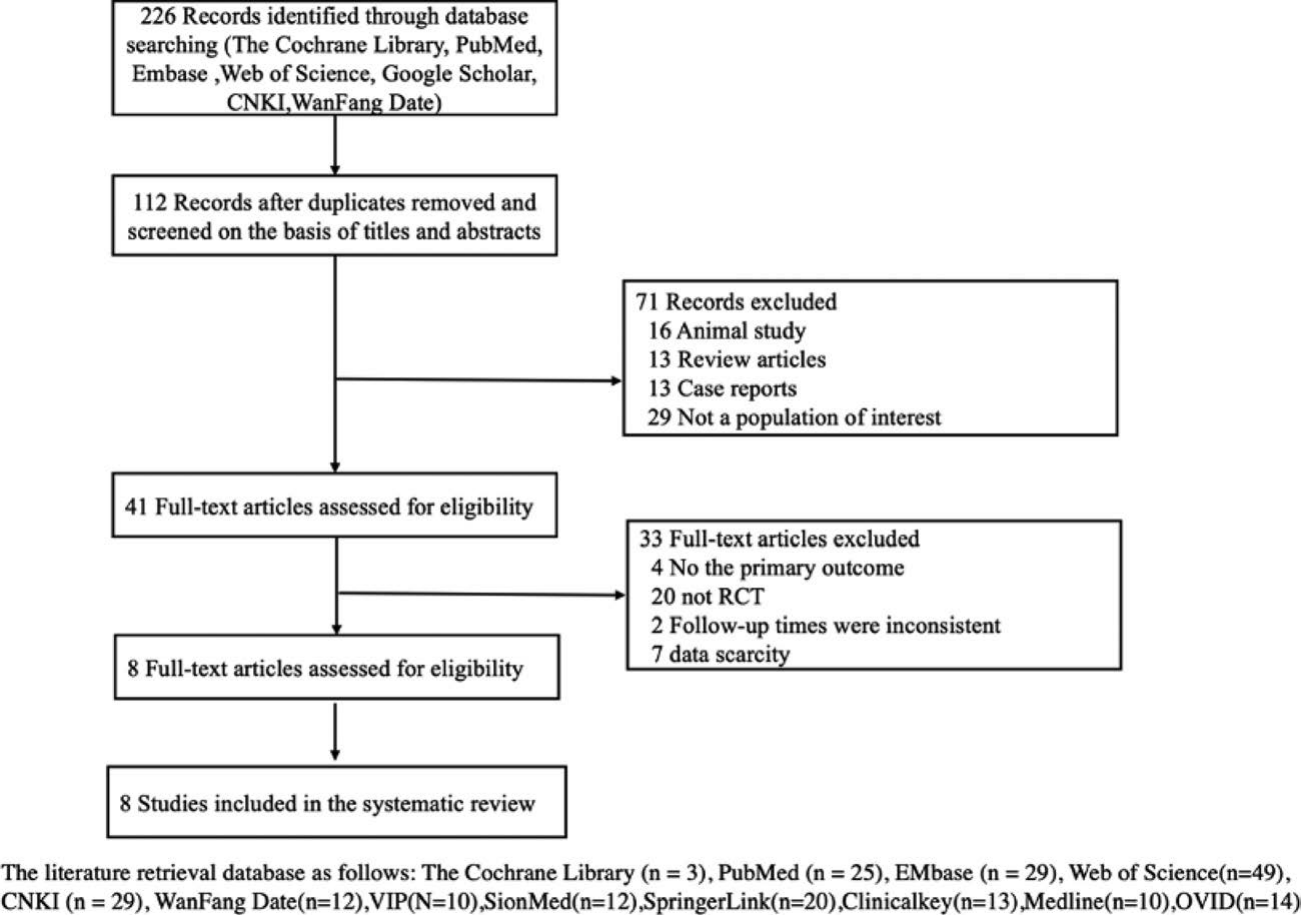
Search and screening flowchart.

After inclusion and exclusion screening and reducing the repeated synthesis of the same control group and the same study population, 8 studies were finally included in the meta-analysis. The screening flowchart is shown in Figure 1.

### 3.2. Basic characteristics of the included studies

Among the 8 studies, 2 studies^[16,17]^ were conducted in Europe; 2 studies^[3,18]^ were conducted in Asia; and the others^[6,19,20]^ were from the Americas and Oceania. Patients in all studies were older than 42 years and were followed for 3 months to 20 years (Table 1).

**Table 1:** Basic characteristics of the included literature.

Author	Year	Type	Area	Sample size	Male (%)	Age	AMD	Follow-up time	Quality assessment (NOS)	OR/RR/HR	95% CI	*P*
Pollack	1996	Cohort study	Asia	47	42.5	80.4 (67‐94)	Early AMD	1 year	8	RR = 4.500	1.027‐19.726	.054
Lintje Ho	2008	Cohort study	Europe	454/11548	40.3/42.9	>55	AMD	5.7 year	9	OR = 1.26	0.85‐1.86	NR
							Early AMD			OR = 1.31	0.88‐1.95	
							Dry AMD			OR = 3.44	1.68‐7.08	
							Wet AMD			OR = 0.93	0.35‐2.49	
Jie Jin Wang	2003	Cohort study	Oceania, North America	6019	42.9	43‐86	Late AMD	5 year	9	OR = 5.7	2.4‐13.6	NR
							Dry AMD			OR = 4.5	1.4‐14.7	
							Wet AMD			OR = 4.9	1.9‐12.4	
Sudha Cugati	2006	Cohort study	Oceania	1952	39.95	>49	Early AMD	10 year	8	OR = 1.25	0.69‐2.25	NR
							Late AMD			OR = 3.31	1.11‐9.87	
							Dry AMD			OR = 2.34	0.51‐10.8	
							Wet AMD			OR = 3.42	1.07‐10.91	
Jau-Der Ho	2018	Cohort study	Asia	3465/10395	45.5/45.5	70.2 ± 9.6	Wet AMD	5 year	9	HR = 2.68	1.55‐4.66	<.01
Helena	2005	Cohort study	Europe	359	36.2	82.4 ± 4.63	AMD	14 year	8	OR = 1.3	0.7‐2.4	NR
Buch							Late AMD			OR = 1.6	0.8‐3.2	
Emily	2009	Cohort study	America	6037	NR	55-80	Wet AMD	5 year	9	OR = 0.76	0.44‐1.30	.31
Y. Chew							Dry MD			OR = 0.55	0.31‐0.99	.047
Jie Jin	2012	Cohort study	Oceania	1711/348	NR	>65	Late AMD	3 year	8	OR = 0.74	0.23‐2.36	NR
Wang							Early AMD			OR = 1.07	0.74‐1.65	
Jie Jin	2016	Cohort study	Oceania	2029	39.8	>65	Late AMD	5 year	8	OR = 0.7	0.4‐1.2	NR
Wang							Early AMD			OR = 0.7	0.5‐1.1	
Ronald	2002	Cohort study	North America	2764	NR	43‐86	AMD	10 year	8	RR = 1.97	1.29‐3.02	<.01
Klein							Early AMD			RR = 1.36	0.82‐2.23	.23
							Late AMD			RR = 3.81	1.89‐7.69	<.01
							Wet AMD			RR = 4.31	1.71‐10.9	<.01
							Dry AMD			RR = 3.18	1.33‐7.60	<.01
Klein,	2012	Cohort study	North America	3275	NR	43‐86	Early AMD	20 year	9	OR = 1.06	0.81‐1.38	.7
B. E.							Late AMD			OR = 1.96	1.28‐3.02	.002
							Early AMD			OR = 1.73	0.93‐3.21	.08
							Late AMD			OR = 2.80	1.03‐7.63	.04
Ronald	1998	Cohort study	North America	3684	NR	43‐86	Early AMD	-	8	OR = 1.73	0.93‐3.21	.08
Klein							Late AMD			OR = 2.80	1.03‐7.63	.04
							Wet AMD			OR = 1.67	0.39‐7.18	.49
							Dry AMD			OR = 3.49	0.80‐15.16	.1
							AMD			OR = 2.57	1.61‐4.11	<.001

NA = not reported.

### 3.3. Quality assessment

Cohort studies were assessed using the cohort study coding manual. If the NOS score was equal to or higher than 8 points, the article was considered high quality. All articles were high-quality literature (Table 1).

### 3.4. Efficacy analysis

**Figure 2.**
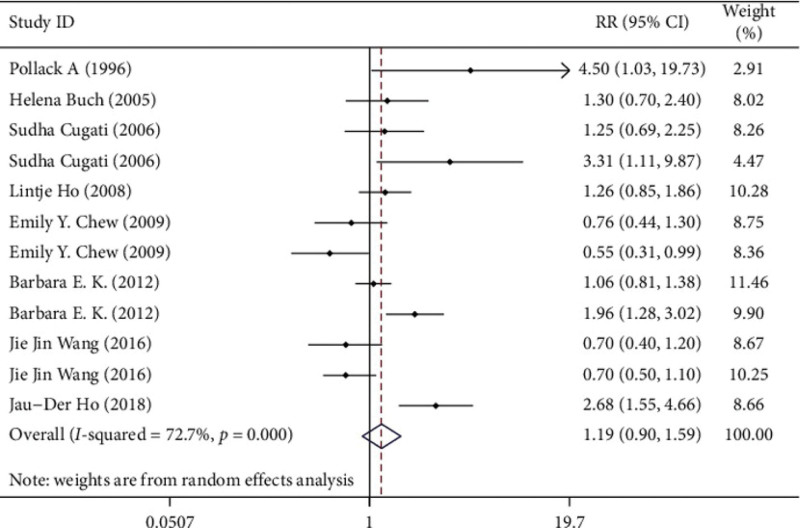
Relationship between cataract surgery and age-related macular degeneration.

There was no significant publication bias in the studies (Egger test *P* = .323; Begg test *P* = .373). The data had high heterogeneity (*I*^2^ = 72.7%). In the random-effects model, cataract surgery and progression of AMD were not significantly associated (RR 1.194, 95% CI 0.897‐1.591), and the difference was not statistically significant (Z = 1.21, *P* = .225) (Fig. 2).

### 3.5. Sensitivity analysis

**Figure 3.**
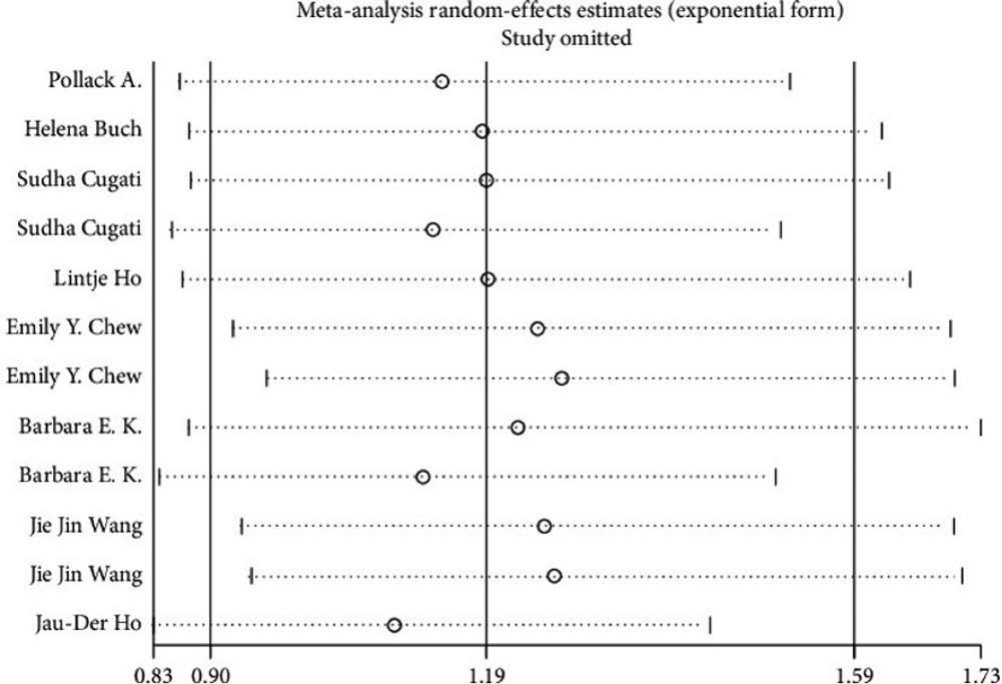
Sensitivity analysis.

**Figure 4.**
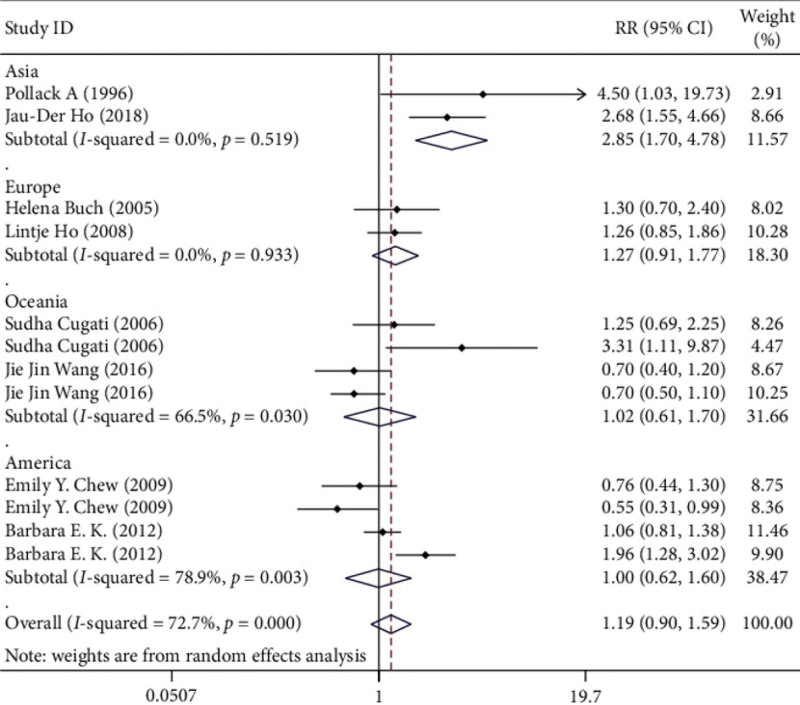
Subgroup analysis (region).

**Figure 5.**
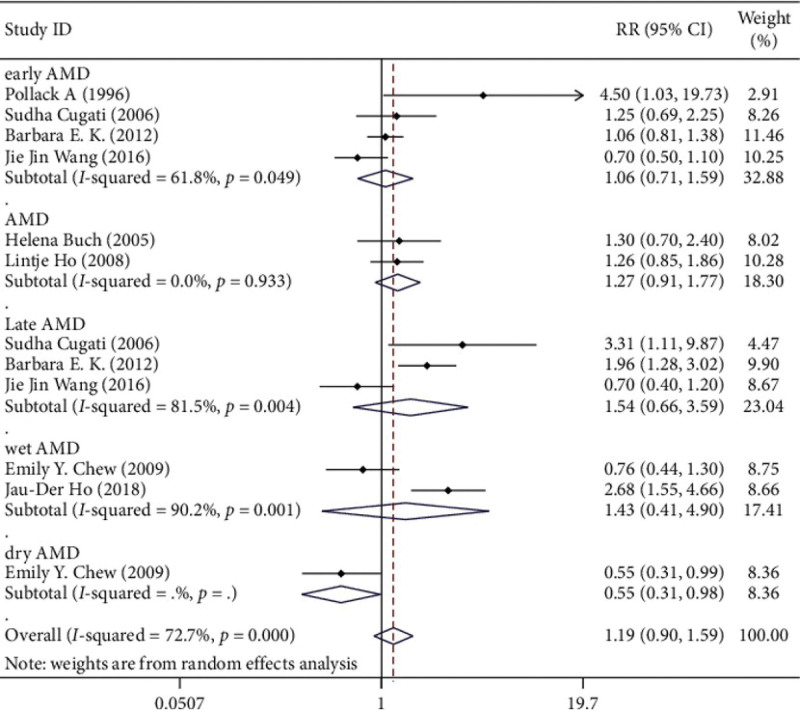
Subgroup analysis (age-related macular degeneration type).

Sensitivity analysis indicated that the sensitivity was low, and the results were relatively stable and credible (Fig. 3). We excluded the effects of the included studies on outcomes one by one, and found that none of them would lead to the reversal of results or significant increase in heterogeneity. The subgroup analysis can be divided into four groups by region, namely, Asia, Europe, Oceania, and America. Asia RR 2.855 (95% CI 1.704‐4.781), Europe RR 1.271 (95% CI 0.914‐1.769), Oceania RR 1.017 (95% CI 0.607‐1.703), Americas RR 0.997 (95% CI 0.621‐1.601) (Fig. 4). Cataract surgery in Asia was significantly associated with AMD progression by subgroup analysis (Fig. 5).

**Figure 6.**
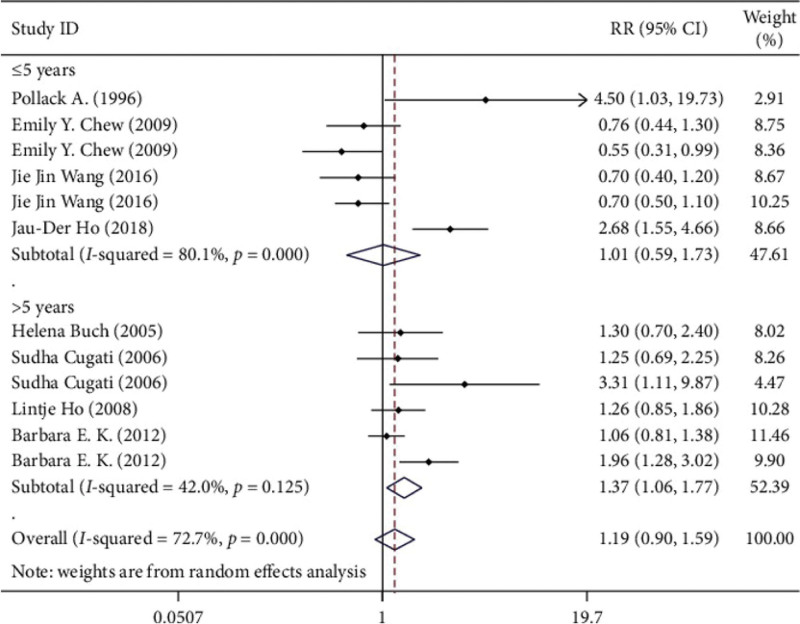
Subgroup analysis (follow-up time).

Grouped by the length of follow-up, the RR of the group less than or equal to 5 years was 1.011 (95% CI 0.592‐1.728), and the RR of the group greater than 5 years was 1.372 (95% CI 1.062‐1.772). The association between cataract surgery and AMD progression became more pronounced with increasing follow-up time (Fig. 6).

## 4. Discussion

Many large-scale epidemiological studies have not clearly identified whether cataract surgery is such an intervention can increase the risk of worsening of AMD progression. Our results show that worsening of AMD progression after cataract surgery are the most common in Asia patients, which is similar to Wang JJ’s study published in 2012,^[20]^ it showed that the incidence of neovascular AMD within 5 years after cataract surgery was 2‐3 times higher than that of nonsurgical patients (HR 2.68; 95% CI 1.55‐4.66; *P* < .001). In a cross-sectional study of North Africans, Lazreg et al^[12]^ found that patients with cataract surgery were more likely to develop AMD than those without surgery (OR: 2.69; 95% CI 1.96‐3.70; *P* < .0001). Darker iris color can increase the risk of worsening of AMD progression too.^[21]^

We divided AMD into early AMD and advanced AMD (including wet AMD and dry AMD) thought severity, and it was found that cataract surgery did not exacerbate all types of AMD.

The studies included differed significantly in the duration of follow-up, and no selection date was proposed. When the follow-up time was >5 years, there was a significant positive association between cataract surgery and increase the risk of worsening of AMD progression. Studies have found that the risk of neovascular AMD differs in different clinical subtypes of AMD. Combining the findings of Beaver Dam and Blue Mountain, cataract surgery was found to be associated with an increased 5-year incidence of neovascular AMD.^[22]^ Klein et al also found that the OR value of advanced AMD is higher in patients with cataract surgery more than 5 years.^[23]^ Epidemiological studies have provided the incidence of AMD after up to 20 years of follow-up, while clinical trials have been followed for no more than 1 year.

In general, the studies included varied widely in study populations and study durations, which were limited in comparability. However, this study included only cohort studies, and the association between cataract surgery and AMD progression became stronger with follow-up beyond 5 years. Many studies with long-term follow-up of AMD patients found that patients who underwent cataract surgery had a higher risk of AMD progression than those who did not.^[6,24]^ In a cross-sectional study combining 3 population studies, those who reported surgery at 5 years or more had a 2.1-fold (95% CI 1.0 vs 4.6) chance of developing advanced AMD compared with those who had surgery less than 5 years. The odds of developing advanced AMD were slightly increased, but the increase was not statistically significant (OR 1.4, 95% CI 0.7 2.6).^[25]^ A population-based cohort of older Australians reported that the long-term (10-year) risk of developing advanced AMD in the eye undergoing surgery was significantly higher than baseline.^[6]^ Ferris et al^[26]^ found that the risk of advanced AMD progression in patients with moderate AMD was as high as 50% within 5 years. In conclusion, AMD patients need regular fundus examinations no matter with or without cataract surgery.

Both cataracts and AMD are strongly age-related, and multiple studies have found that cataracts and AMD may share the same epidemiological risk factors, but this study did not find that cataract surgery and the risk of worsening of AMD progression are directly related or have a clear connection.^[27,28]^ Although most of the included observational studies did control for different familiar confounders, but cataract status is still residual confounding for the possible interactions of AMD, cataract, and cataract surgery do not all appear to be consistent. However, all of these are insufficient to explain the association between cataract surgery and the risk of worsening of AMD progression in this study.

This review has the following limitations: First, Persuading cataract patients to randomize surgery is difficult, so it’s hard to conduct randomized controlled trials (RCTs),which with the highest level of evidence, prospective or retrospective studies can provide the most powerful evidence under the circumstances with a series of obstacles of RCTs.^[28]^ In addition, there are some less standardized studies, the incidence of AMD was artificially reduced for some patients with unclear fundus cataracts cause were excluded. Therefore, all those may affect the statistical analysis of the association of AMD with other parameters.

In conclusion, there is still a significant positive correlation between cataract surgery and increase the risk of worsening of AMD progression, and faster progression of early-to-late AMD found in cataract surgery with longer follow-up of patients. However, based on the result of this review, we cannot draw conclusions about the effect of cataract surgery on the risk of worsening of AMD progression. In conclusion, the studies we included were highly heterogeneous in terms of study population and study period, and were highly heterogeneous, so their comparability was limited. more clinical trials (with sufficient statistical power) are needed, which should ideally be able to adequately control for confounding variables such as age and cataract severity, and subgroup analyses can be set up to make the study more precise Regarding the need for more clinical trials (with sufficient statistical power) to demonstrate this hypothesis by adequately controlling for confounding variables such as age and cataract severity.

## Author contributions

ZYC conceived and designed the experiments. YZ and FYT performed the experiments and data analysis. YZ and ZYC provided the reagents, materials and analysis tools. ZYC wrote the manuscript. ZYC revised the work critically for important intellectual content. All authors have read and approved the final version of the manuscript.

**Conceptualization:** Zhaoyan Chen.

**Data curation:** Zhaoyan Chen.

**Investigation:** Ya Zeng.

**Methodology:** Ya Zeng.

**Project administration:** Fangyuan Tian.

**Resources:** Fangyuan Tian.
